# *Chlamydia trachomatis, Neisseria gonorrhoeae* and syphilis among men who have sex with men in Brazil

**DOI:** 10.1186/s12889-015-2002-0

**Published:** 2015-07-21

**Authors:** Cynthia B. Cunha, Ruth K. Friedman, Raquel B. de Boni, Charlotte Gaydos, Maria R.C. Guimarães, Brenda H. Siqueira, Sandra W. Cardoso, Leonardo Chicayban, José R. Coutinho, Carolyn Yanavich, Valdilea G. Veloso, Beatriz Grinsztejn

**Affiliations:** Laboratory of Clinical Research in STD/AIDS, Evandro Chagas National Institute of Infectious Diseases (INI-Fiocruz), Rio de Janeiro, Brazil; Division of Infectious Diseases, John Hopkins University, Baltimore, USA; Av. Brasil, 4365, CEP 21040-360 Rio de Janeiro, Brazil

**Keywords:** **S**exually transmitted diseases, Resource limited settings, HIV prevention, Rectal chlamydia, Rectal gonorrhea, Nucleic acid amplification tests (NAAT), Brazil

## Abstract

**Background:**

Sexually transmitted diseases (STD) are frequently asymptomatic and increase the likelihood of transmitting and acquiring HIV. In Brazil, the guidelines for STDs diagnosis and treatment are based on the syndromic approach. Nucleic acid amplification tests (NAAT*)* has been recommended as routine STDs screening in some countries, especially for men who have sex with men (MSM). Limited data are available about how to best define target groups for routine screening by NAATs within this population. We aimed to assess the prevalence of rectal and urethral *Chlamydia trachomatis* (CT) and *Neisseria gonorrhoeae* (NG) infections and syphilis, and the factors associated with having at least one STD among HIV-infected and uninfected MSM in Rio de Janeiro, Brazil.

**Methods:**

From August 2010 to June 2012, 391 MSM were enrolled into the Evandro Chagas National Institute of Infectious Diseases-INI-Fiocruz cohort, and 292 MSM (HIV-infected:211 and HIV-uninfected:81) were included in this study. NAATs were performed on the rectal swabs and urine for CT and NG. The rapid plasma reagin test and microhemagglutination assay for *Treponema pallidum* were performed for syphilis diagnosis.

**Results:**

The overall prevalence of STD was 20.0 % (95%CI:15.7-25.1): 10 % anorectal chlamydia; syphilis 9.9 %; anorectal gonorrheae 2.5 %; and urethral chlamydia 2.2 %; no case of urethral gonorrheae was detected. The proportion of HIV-positive MSM who had at least one STD was nearly two times that of HIV-negative MSM (22.6 % vs 13.2 %; P = 0.09). The frequency of each STD, except for anorectal NG (1.5 % vs.5.2 %), was higher among HIV-positive than HIV-negative individuals. Among the 211 asymptomatic participants, 17.5 % (n = 37) were identified as having at least one STD; 10.4 % (n = 22/211) tested positive for anorectal chlamydia. Sixty five percent of HIV-positive MSM were asymptomatic at the time of the STD diagnosis, while 100.0 % of the HIV-negative MSM. Age (APR = 0.78; 95%CI:0.60-1.00 for each additional ten years) and a positive-HIV serostatus (APR = 2.05; 95%CI:1.03-4.08) were significantly associated with STD diagnosis.

**Conclusion:**

An overall high STD-prevalence rate was observed, especially among HIV-infected and in younger individuals, and the majority of STDs were asymptomatic. STD screening using NAATs among asymptomatic MSM is a potentially cost-effective intervention for the prevention of HIV infection among MSM.

## Background

In developed settings, the prevalence of sexually transmitted diseases (STDs) among gay and other men who have sex with men (MSM) has been on an upward trend since the late 1990s [1]. In low- and middle-income countries STDs are a significant burden to the health care system [2], further fueled by a lack of systematic surveillance and population-based studies of STD prevalence. In South America, including Brazil, the proportion of new HIV infections is on the rise in young gay and other MSM [3], making this population particularly vulnerable to other sexually transmitted diseases (STDs) [4].

There are several factors, such as the number of sexual partners [5], inconsistent condom use [6] and HIV infection [7] that are associated with increased prevalence of STDs among MSM. Individuals infected with HIV are more susceptible to other STDs because they are immunocompromised and less capable of mounting a protective response against sexually transmitted pathogens [8, 9]. STDs are frequently asymptomatic, therefore, increasing the likelihood of transmitting and acquiring HIV [10].

Because targeted groups within this population are not well defined or studied, routine STD screening should be recommended for all MSM [11]. In Brazil, STD clinical management relies mostly on the syndromic approach, as molecular STD diagnosis is not routinely available in the Public Health System [12].

This study aimed to assess the prevalence of rectal and urethral CT and *Neisseria gonorrhoeae* (NG) infections and syphilis, as well as to describe the factors associated with a positive diagnosis of at least one STD among HIV-infected and uninfected MSM at a referral center for MSM health in Rio de Janeiro, Brazil.

## Materials and Methods

A cross-sectional analysis, nested within a cohort study was conducted at Evandro Chagas National Institute of Infectious Diseases-INI-Fiocruz (formerly known as the Evandro Chagas Clinical Research Institute-IPEC), Rio de Janeiro, Brazil. The parent cohort study was designed to evaluate the prevalence and incidence of anal HPV infection and intraepithelial anal lesions among HIV infected and uninfected men [13]. In summary, men were recruited by trained staff either while in the clinic waiting room or by phone. Inclusion criteria for the parent cohort study were: being aged 18 years or older, not having anal cancer or related treatments (surgery, radiotherapy, and chemotherapy) and a willingness to sign the informed consent form. The exclusion criteria were: the use of immunomodulators agents, such as prednisone (dosage > 10 mg/day), interleukin or interferon. HIV positive men were already under care at the INI HIV Clinic, and HIV negative men attending the clinic for HIV prevention services. At the enrollment visit, information on demographic, sexual behavior, substance use as well as history of prior STDs were collected using Audio Computer Assisted Interview (ACASI) and specimens for STD diagnostic testing were collected. The study population for the present analysis were men who have sex with men (MSM), herein defined as men that reported having at least 1 male sexual partner within the past 12 months (regardless of having female partners).

**Fig. 1:**
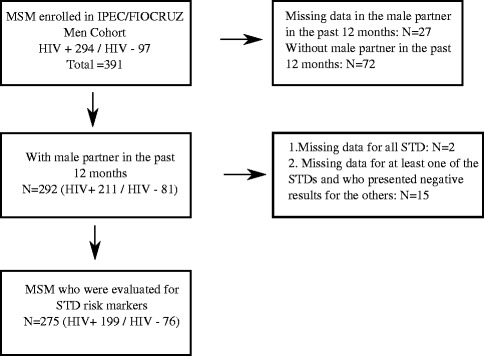
Study population- INI/Fiocruz, 2010–2012

From August 2, 2010 to June 30, 2012, 391 MSM were enrolled into the parent cohort study. Of these, 72 (18.4 %) were excluded for not having a male sexual partner(s) within the last 12 months, and 27 (6.9 %) were excluded because of missing information regarding their sexual partners within the past 12 months (Fig. 1). In total, 292 MSM were included in this analysis. To determine the prevalence for each specific STD, we considered all participants who had available results for each of the STDs under evaluation, thus explaining the different denominators. The outcome “Having one or more STD at study entry” was defined as having a positive testing for at least one of the following STDs: CT (rectal, urethral, or urine), NG (rectal, urethral, or urine) or syphilis, at the study baseline visit. If a participant tested positive for at least one of the STDs under evaluation, regardless of having missing data on other STD tests, his data contributed to the analysis of the outcome “Having one or more STD at study entry”. Thus, 275 men were included in the analysis of the associated factors of having at least one STD (Fig. 1).

### STD diagnosis

Rectal CT and NG infection were diagnosed using APTIMA Combo 2 assay (Gen-Probe/Hologic San Diego, CA). Testing was processed at the Johns Hopkins STD Laboratory, in Baltimore, Maryland, USA. All indeterminate results for rectal CT/NG were repeated using the same tests on the same sample. If the repeated test was conclusive, the results were reported accordingly. If remained indeterminate, the result was reported as negative. Urethral CT and NG infection were diagnosed using urine samples on the Abbott RealTi*m*e platform and the NG/CT Amplification Reagent Kit (Abbott Molecular, Des Plains, IL); these samples were processed at the INI-Fiocruz Laboratories.

The rapid plasma reagin (RPR) test was performed for syphilis screening; positive results were confirmed using a microhemagglutination assay for *Treponema pallidum* (MHA-TP). Titers equal to or higher than 1/8 and a positive MHA-TP constituted a syphilis diagnosis. HIV infection was diagnosed according to the Brazilian HIV diagnosis algorithm (www.aids.gov.br). HIV-negative participants were tested at the baseline visit and also at each follow-up visit. Both the INI-Fiocruz Laboratories and the Johns Hopkins University STD Research Laboratory successfully participate in the College of American Pathologists (CAP) External Quality Assurance (EQA) proficiency testing panels for all relevant testing associated with this study.

### Measures

*STD-related symptoms* included urethral/anal discharge, anal or genital nodules, anal or genital ulcers, spontaneous anal pain, tenesmus and anal pruritus. Individuals were considered symptomatic when at least one of the above-referenced symptoms was reported.

*Demographic variables* included age, self-reported skin color (further classified as white or non-white) and schooling (years of formal education).

The *number of male and female sexual partners* within the last 12 months was determined using the following questions: “During the past 12 months, how many men and how many transvestite/transsexual/transgender(s) did you have sex with?” and “During the past 12 months, how many women did you have sex with?” The latter question was categorized as “none” or “at least one female partner”.

*Stable partner in the past 3 months* was defined as “Yes” if the participant considered at least one sexual partner within the last 3 months to be a stable partner (husband, wife, boyfriend or girlfriend).

*HIV-positive male sexual partner in the past 3 months* was determined using the following questions: a) “In the past 3 months, how many HIV-positive male partners did you have insertive anal sex with?” and b) “In the past 3 months, how many HIV- positive male partners did you have receptive anal sex with?”.

Each study participant provided information, if known, on the HIV serostatus of his sexual partners. Independent HIV testing was not performed on the partners. *Alcohol and stimulant use before or during sex* were determined using the following questions: “In the past 3 months, were you drunk or high before or during sex?" and “In the past 3 months, did you use either inhaled or intravenous illicit drugs before or during sex?” [14].

*Commercial sex within the past 3 months* was defined as the exchange of sex for money/other favors and/or seeking commercial sex workers. This was determined using the following questions: “In the past 3 months, did you have sex for money, drugs or other favors?” and “In the past 3 months, did you look for commercial sex workers?”.

*Anal sexual practices with male partners during the past 3 months* were evaluated using the following questions: a) “In the past 3 months, how many HIV-negative male partners did you have insertive anal sex with?”; b) “In the past 3 months, how many male partners, with an unknown HIV-serostatus did you have insertive anal sex with?”; c) “In the past 3 months, how many HIV-negative male partners did you have receptive anal sex with?”; and d)“In the past 3 months, how many male partners, with an unknown HIV-serostatus did you have receptive anal sex with?” The answers were categorized as ‘only insertive’, ‘only receptive’ or ‘both’.

*Unprotected anal intercourse during the past 3 months* was defined as any positive answer to the following questions: “In the past 3 months how many: a) HIV-negative male partners did you have insertive anal sex with, without using condoms? b) male partners, with an unknown HIV-serostatus did you have insertive anal sex with, without using condoms? c) HIV-negative male partners did you have receptive anal sex with, without using condoms? and d) male partners, with an unknown HIV-serostatus, did you have receptive anal sex with, without using condoms?”.

### Statistical analysis

For those with results available we have described the overall prevalence of CT, NG and syphilis. These results are sorted out based on HIV status. The overall prevalence for *having one or more STD at study entry’* outcome and the confidence interval for the proportion [15] were calculated and are also presented sorted to HIV status using Chi-square test for comparison. Generalized linear models using logarithmic linkage and Poisson distribution with robust variance were used to estimate the prevalence ratio between selected variables and the outcome [16]. Age, schooling, skin color, number of male sexual partners within the previous 12 months, having female partners, having a stable partner, having an HIV-positive sexual partner, having been high from alcohol and having used stimulants before/during sex, reporting commercial sex, position during anal sex, unprotected anal sex with male partner (s) in the last 3 months and HIV status were evaluated in the univariate analysis. Variables that were associated with an STD according to the univariate analysis (P < 0.25) were entered into the initial multivariate model. Multi-colinearity was tested using generalized colinearity diagnostics (GVIF). Variables were kept in the final multivariate model if (a) the P value < 0.05 or (b) it was a confounder, e.g., when removed, a change equal or higher than 10 % in the prevalence ratio of any other variable of the model was observed [17]. We forced the variable age (a priori) into the multivariate models. The STATA/SE 10.1 software was used to perform the analysis.

### Ethics

The study was approved by the IPEC-FIOCRUZ IRB (CAAE 0044.0.009.000-09); all study participants signed an informed consent prior to cohort enrollment.

## Results

Overall 292 participants were included in this analysis, 211 HIV-infected and 81 HIV-uninfected. The HIV-infected participants were significantly older than the HIV-uninfected [median (IQR): 39.0 (31.0–46.0) vs 33.0 (27.0–40.0); P = 0.000], more frequently self-reported as white (53.4 % vs 27.5 %; P < 0.0001), had a lower number of male sexual partners within the previous 12 months [median (IQR): 5.0 (2.0–10.0) vs 10.0 (4.0–20.0); P = 0.002], and had less unprotected receptive or insertive anal sex with other men during the past 3 months (35.6 % vs 50.7 %; P = 0.027) Overall, 8.3 % (n = 24/289) of participants were circumcised (7.5 % HIV-infected and 8.6 % HIV-uninfected; P = 0.759). The median time since HIV diagnosis was 75.8 (41.2–162.4) months; 77 % (n = 161/209) of the HIV-infected participants were using cART at study entry; the median CD4 cell count (IQR) was 555.0 (375.0–789.5) cells/mm3 with 7.8 % (n = 15/192) having less than 200 cells/mm3; 37.1 % (75/202) had a detectable HIV viral load. All study participants consented to STD screening including the genital swabbing procedures.

### STD prevalence

Table 1 shows the prevalence of STD, both overall and individual, stratified by HIV status. The most frequently diagnosed STDs were rectal Chlamydia (10.0 %) and Syphilis (9.9 %), while urethral Chlamydia (2.2 %) and Rectal Gonorrhea (2.5 %) were less commonly reported. Among MSM who participated in the analysis of associated factors to have at least one STD, 199 and 76 were HIV-infected and uninfected MSM, respectively. A total of 20.0 % (95%CI: 15.7–25.1) had at least one STD, not including HIV infection. The proportion of HIV-positive MSM who had at least one STD (N = 45/199) was nearly 1.7 times that found for the HIV-negative MSM (N = 10/76) (22.6 % vs 13.2 % *P* = 0.09). A single STD was detected in 14.7 % (n = 43) participants and 4.1 % (n = 12) were diagnosed with two STDs. The frequency of each STD, except for anorectal NG, was higher among HIV-positive than HIV-negative individuals.

**Table 1:** Prevalence of sexually transmitted diseases among MSM-INI-Fiocruz, Rio de Janeiro, Brazil

	Gonorrhea		Chlamydia		Syphilis	At least one STD
	Urethral	Rectal	Urethral	Rectal		
	(N = 273)^a^	(N = 279)^a^	(N = 273)^a^	(N = 279)^a^	(N = 284)^a^	(N = 275)^b^
N (%)	-	7 (2.5)	6 (2.2)	28 (10.0)	28 (9.9)	55 (20.0)
HIV serostatus. N (%)						
Positive^c^	-	3 (1.5)	6 (3.0)	24 (11.9)	22 (10.8)	45 (22.6)
Negative^d^	-	4 (5.2)	-	4 (5.2)	6 (7.5)	10 (13.2)

^a^Different denominators are explained by availability of results for each STD evaluated

^b^Participants who tested positive for at least one STD, regardless of having missing information for other STD, were included and considered as an event. HIV-infected MSM = 199; HIV-uninfected MSM = 76

^c^Number of HIV-infected MSM according to the availability of results for each STD: urethral NG = 197; anorectal NG = 202; urethral CT = 197; anorectal CT = 202; Syphilis = 204; At least one STD = 199

^d^Number of HIV-uninfected MSM according to availability of results for each STD: urethral NG = 76; anorectal NG = 77; urethral CT = 76; anorectal CT = 77; Syphilis = 80; At least one STD = 76

### STD symptoms

Information on STD symptoms was available for 263 participants, with 211 (80.2 %) being asymptomatic and 52 (19.8 %) reported having symptoms (Table 2). Anorectal symptoms were reported by 10.9 % of participants, with rectal discharge (3.4 %), anal nodules (4.1 %), and anal ulcers (5.2 %) being the most frequently reported symptoms. Of the 32 participants with anorectal CT or NG infection, 9.4 % had anorectal symptoms; 1.5 % and 2.3 % of the participants reported having genital ulcers or nodules, respectively. No urethral discharge was reported. Among MSM with syphilis, 13 % presented rectal or genital ulcers.

**Table 2:** Proportion of at least one STD^a^ among symptomatic^b^ and asymptomatic MSM, INI/Fiocruz, Rio de Janeiro,Brazil

At least one STD	Symptomatic^b^, n (%)	Asymptomatic, n (%)
Yes	15 (28.8)	37 (17.5)
No	37 (71.2)	174 (82.5)
Total	52 (100.0)	211 (100.0)

^a^At Least One STD = Chlamydia/Gonoccocal urethral and/or anal infection or Syphilis

^b^Syntomatic = report of uretrhal/anal discharge, anal or genital nodule, anal or genital ulcer, spontaneous anal pain, tenesm and, anal pruritis

Among the 211 asymptomatic participants, 17.5 % (n = 37) were identified as having at least one STD: rectal gonorrhea in 2.8 %, urethral chlamydia in 1.5 %, rectal chlamydia in 10.4 %, and syphilis in 5.7 %. 65 % of the HIV-infected MSM and 100 % of the HIV negative MSM were asymptomatic at the time of the STD diagnosis.

### Participants’ characteristics according to STD diagnosis

The characteristics of these participants are described in Table 3 and are sorted by STD infection status. For those participants with at least one STD (n = 55), the median age was 35.0 years (IQR 29.0–42.0); approximately 20 % had less than eleven years of schooling; 53.7 % self-reported to be non-white. The median number of male sexual partners in the last 12 months was 8.0 (IQR 3.0–27.0).Within the last 3 months before entering the study, 32.7 % participants had an HIV-positive partner; 20.8 % used stimulants before or during sex; 46.3 % practiced commercial sex, and almost 60 % reported both insertive and receptive anal sex. Almost half of the HIV-infected MSM diagnosed with an STD had a detectable plasma HIV viral load.

**Table 3:** Characteristics of MSM according to sexually transmitted disease. INI/FIOCRUZ, Rio de Janeiro, Brazil

Characteristics	Sexually Transmitted Disease – N(%)		
	No	Yes	Total
			N = 275
Age^1^	38.0 (30.0-46.0)	35.0 (29.0-42.0)	38.0 (30.0-45.0)
Less than 11 years of schooling	50 (23.1)	12 (22.2)	62 (23.0)
Skin color – Non White	117 (53.7)	29 (53.7)	146 (53.7)
No. of male sexual partners in the last 12 months^1^	5.0 (2.0-14.0)	8.0 (3.0-27.0)	5.0 (2.0-15.0)
Had a female sexual partner in the last 12 months	23 (10.5)	7 (12.7)	30 (10.9)
Had a stable partner in the last 3 months	100 (46.9)	25 (47.2)	125 (47.0)
HIV-positive male sexual partner in the last 3 months	48 (23.1)	17 (32.7)	65 (25.0)
High from alcohol use before/during sex in the last 3 months	120 (56.3)	29 (54.7)	149 (56.0)
Stimulant use before/during sex in the last 3 months	28 (13.1)	11 (20.8)	39 (14.7)
Commercial sex in the last 3 months	86 (40.4)	25 (46.3)	111 (41.6)
Anal intercourse with men in the last 3 months			
Only insertive	34 (16.3)	8 (15.4)	42 (16.2)
Only receptive	43 (20.7)	5 (9.6)	48 (18.5)
Both	101 (48.6)	30 (57.7)	131 (50.4)
None sexual partner	30 (14.4)	9 (17.3)	39 (15.0)
Unprotected anal sex with men in the last 3 months	80 (38.5)	23 (44.2)	103 (39.6)
HIV infected	154 (70.0)	45 (81.8)	199 (72.4)
Time since HIV diagnosis, months (N = 185)			
Median (IQR)	75.4 (40.3-163.8)	85.4 (42.0-161.9)	78.5 (41.2-162.4)
<12	6 (4.1)	1 (2.6)	7 (3.8)
12-36	28 (19.2)	8 (20.5)	36 (19.5)
>36	112 (76.7)	30 (76.9)	142 (76.8)
Currently receiving cART (N = 197)	122 (80.3)	33 (73.3)	155 (78.7)
Current CD4 (N = 183)			
Median (IQR)	542.0 (359.0-781.0)	597.5 (425.0-803.0)	554.0 (376.0-781.0)
<200	11 (7.7)	3 (7.5)	14 (7.7)
200-350	23 (16.1)	4 (10.0)	27 (14.8)
>350	109 (76.2)	33 (82.5)	142 (77.6)
Total	143 (100.0)	40 (100.0)	183 (100.0)
HIV viral load (N = 193)			
Indetectable	99 (65.6)	23 (54.8)	122 (63.2)
Detectable	52 (34.4)	19 (45.2)	71 (36.8)
Total	151 (100.0)	42 (100.0)	193 (100.0)

Missing data: STD = 5.8 %, Years of schooling = 3.1 %, Skin color = 2.1 %, Female sexual partner in the last 12 months = 4.1 %, Stable partner in the last 3 months = 3.4 %, HIV-positive male sexual partner in the last 3 months = 5.8 %, High from alcohol use before/during sex in the last 3 months = 3.4 %, Stimulant use before or during sex in the last 3 months = 3.4 %, Commercial sex in the last 3 months = 3.1 %, Anal intercourse with men in the last 3 months = 5.8 %, Unprotected anal sex with men in the last 3 months = 5.8 %, Time since HIV diagnose = 7.0 %, Currently receiving cART = 1.0 %, Current CD4 = 8.0 %, HIV viral load = 3.0 %. ^1^Median (IQR)

### Factors associated with STD diagnosis

In the univariate analysis, besides age and HIV serostatus, some sexual risk behaviors were found to be associated with high STD prevalence. For each 10 male sexual partners in the past 12 months, a 1 % increase in STD prevalence was observed (NAPRs = 1.01; 95 % CI: 1.00–1.02). MSM who reported having HIV-positive male sexual partners during the 3 months prior to cohort enrollment had a higher STD prevalence as opposed to those who reported no such partners (NAPRs = 1.46; 95 % CI: 0.88–2.42). Stimulant use before or during sex in the 3 months prior to cohort enrollment (NAPRs = 1.52; 95 % CI: 0.86–2.70) was also associated to a high STD prevalence in the univariate analysis.

In the final multivariate model, age (APR = 0.78; 95 % CI: 0.60-1.00 for each additional ten years) and HIV infection (APR = 2.05; 95 % CI: 1.03–4.08) remained significantly associated with any STD diagnosis (Table 4).

**Table 4:** Factors associated with sexually transmitted diseases diagnosis among MSM. INI/FIOCRUZ, Rio de Janeiro, Brazil

Characteristics	PR^a^ (CI 95 %)			
	NAPR^b^	P value	APR^c^	P value
Age (each ten years)	0.82 (0.64-1.05)	0.110^d^	0.78 (0.60-1.00)	0.049
Less than 11 years of schooling	0.96 (0.54-1.71)	0.885	-	-
Skin color – Non White	1.00 (0.62-1.62)	0.996	-	-
No. of male sexual partners in the last 12 months (each ten partners)	1.01 (1.00-1.02)	0.095^d^		
Had a female sexual partner in the last 12 months	1,19 (0,59-2,39)	0,623	-	-
Had a stable partner in the in the last 3 months	1.01 (0.62-1.63)	0.977	-	-
HIV-positive male sexual partner in the last 3 months	1.46 (0.88-2.42)	0.146^d^	-	-
High from alcohol use before/during sex in the last 3 months	0.95 (0.58-1.54)	0.832	-	-
Stimulant use before/during sex in the last 3 months	1.52 (0.86-2.70)	0.148^d^	-	-
Commercial sex in the last 3 months	1.21 (0.75-1.95)	0.431	-	-
Anal intercourse with men in the last 3 months (Ref: Only insertive)				
Only receptive	0.55 (0.19-1.55)	0.255	-	-
Both	1.20 (0.60-2.42)	0.606	-	-
None sexual partner	1.21 (0.52-2.83)	0.658	-	-
Unprotected anal sex with men in the last 3 months	1.21 (0.74-1.97)	0.447	-	-
HIV infected	1.72 (0.91-3.24)	0.094^d^	2.05 (1.03-4.08)	0.040

^a^PR = Prevalence Ratio

^b^NAPR = Non-Adjusted Prevalence Ratio

^c^APR = Adjusted Prevalence Ratio; 260 participants were included in the multivariate models; 15/275 (5.4 %) had missing data in at least one covariate

^d^ Variables with P < 0.25 in univariate analysis and were entered in the initial multivariate model

## Discussion

A high STD prevalence rate was observed among the MSM enrolled in this study, particularly anorectal CT (10 %) and syphilis (9.9 %). Because of its potential for facilitating HIV acquisition and transmission, the high prevalence of anorectal CT infection may be a contributing factor to the ongoing HIV epidemic in MSM in our setting. In order to confirm this hypothesis, additional representative, population-based MSM studies are needed.

Our findings are consistent with other reports [18], showing that most anorectal chlamydial and gonoccocal infections as well as syphilis were asymptomatic. Our data emphasize the need to educate MSM, particularly those who are HIV infected, on the topic of STD awareness. Asymptomatic STDs are especially concerning because as the untreated infections persist, the risk of contracting and transmitting HIV increases [19]. Additionally, in the absence of symptoms, the infected individual is not prompted to seek health services or adjust his risk practices [20]. We believe that these data are the first to describe the prevalence of anorectal CT and NG in MSM in Brazil. Our findings illustrate the urgent need to implement STD screening to MSM [11, 21], as they remain the most affected by the HIV epidemic in our country [3].

As seen in other studies, when compared to HIV-negative men, HIV-positive men were more often diagnosed with STD (22.6 % vs 13.2 %) [22–25]. We observed that the HIV-infected MSM in our study had a high prevalence of anorectal CT and syphilis as seen in other settings [26, 27]. The discrepancy between sexual behavior and STD prevalence may be explained by the fact that HIV-positive individuals enrolled in our study were engaged in care and had a median follow-up period of 6.5 years (IQR: 3.4–13.5), therefore receiving positive prevention counselling that might have influenced their sexual risk behavior. Moreover, as STD molecular screening is not standard of care in Brazil, and diagnosis is only done using the syndromic approach, it may be the case that the higher prevalence among the HIV positive MSM was driven by infections acquired prior to their linkage to care, that may have remained undiagnosed and thus, untreated.

In our study, only two variables were associated with an increased risk of a STD diagnosis in the multivariate analysis, younger age and HIV co-infection. Several studies have also found that younger age is associated with an increased risk for a STD diagnosis [7, 28, 29]. This finding is of paramount importance because 40 % of new HIV diagnoses in Brazil are among the very young MSM aged 15–24 years [3]. Our study showed that for every additional ten years of age, the prevalence of having at least one STD decreased by 22 %. These results may contribute to the development of recommendations for targeted routine STD screenings for MSM in our setting.

Potential explanations for the lack of an association with well-accepted variables, such as the number of sexual partners, stimulant use before or during sex in the past 3 months and unprotected sex, are the different methods and measures used across the different studies and the limited sample size. Another explanation may be related to our definition of STD, which combined different STDs into a composite endpoint. For example, different factors may be predictive of syphilis, urethral CT and rectal NG.

Although progress has been made in increasing HIV prevention and treatment services in resource-limited settings, major hurdles remain for the diagnosis of other STDs. As a result the information on CT and GC infection is likely to be grossly underestimated. Because many STDs are asymptomatic, the current clinical standard of relying on sexually active persons to self-detect STD symptoms is an inadequate triage method [30]. Although it is well recognized that routine STD testing for MSM should be incorporated into the Brazilian clinical guidelines, the high cost burden to implement such testing makes it an unattainable goal. More epidemiological, coupled with cost effectiveness studies are urgently needed to better define targeted MSM groups for screening. Our results show that younger MSM and those living with HIV/AIDS are important targets for STD screening. The striking benefit of cART in preventing HIV transmission among heterosexual and MSM serodiscordant couples [29, 30] has paved the way and triggered the expansion of utilizing cART for prevention. Hence, STD co-infections in HIV-infected populations may have significant implications on the expected outcomes of the HIV Test and Treat policy, which was recently adopted by the Brazilian Ministry of Health [31].

Our study has several limitations. First, regardless of HIV serostatus, all participants were under care at our institution. As a result they may have received HIV prevention counseling and may have received STD treatment, prior to enrollment into the parent cohort study, leading to an underestimation of our STD prevalence rate. In addition, although we have used CASI to collect sexual behavior data, we cannot exclude that underreporting of risky sexual risk behavior in HIV-infected MSM was related to social desirability bias. We enrolled a convenience sample at a single site, and data may not be generalizable to MSM in Brazil. In addition, the small sample size precluded the analysis of factors associated with individual STDs.

## Conclusions

In summary, our findings showing a high STD prevalence rate, especially the high proportion of asymptomatic GC and CT infections support the CDC recommendation to offering periodic CT and GC NAAT testing to MSM in developing countries [11]. It poses a challenge to the Brazilian health system that offers only syndromic management of STD irrespective of the population. [32]. Lack of diagnosis of asymptomatic GC and CT infections in a context of an epidemic mostly driven by MSM may compromise the impact of the current test–and-treat strategy on the control of the HIV epidemic in Brazil. Routine GC and CT NAAT testing targeting asymptomatic MSM may be proven to be cost-effective and should be explored in Brazil [33].

## Abbreviations

ACASI: Audio computer assisted interview
APR: Adjusted prevalence ratio
cART: Combined antiretroviral therapy
CT: *Chlamydia trachomatis*
HIV: Human immunodeficiency virus
MHA-TP: Microhemagglutination assay for *Treponema pallidum*
MSM: Men who have sex with men
NAAT: Nucleic acid amplification tests
NG: *Neisseria gonorrhoeae*
RPR: Rapid plasma reagin
STD: Sexually transmitted diseases.
